# Expression of LIM domain-binding 3 (LDB3), a striated muscle Z-band alternatively spliced PDZ-motif protein in the nervous system

**DOI:** 10.1038/s41598-023-27531-5

**Published:** 2023-01-06

**Authors:** Yotam Blech-Hermoni, Kalpana Subedi, Maya Silver, Leah Jensen, Stephen Coscia, Malcolm M. Kates, Yongmei Zhao, Castle Raley, Nancy Edwards, Bao Tran, Abhik Ray-Chaudhary, Pankaj Pathak, Ami Mankodi

**Affiliations:** 1 Neurogenetics Branch, National Institute of Neurological Disorders and Stroke, National Institutes of Health, Bethesda, MD USA; 2 Frederick National Laboratory for Cancer Research, Leidos Biomedical Research, Inc., Frederick, MD USA; 3 Surgical Neurology Branch, National Institute of Neurological Disorders and Stroke, National Institutes of Health, Bethesda, MD USA; 4 Neurogenetics Branch, National Institute of Neurological Disorders and Stroke, National Institutes of Health, 35 Convent Drive, Building 35, Room 2A-112, Bethesda, MD 20892-3705 USA; 5 Present Address: Milken Institute School of Public Health, George Washington University, Washington, DC USA

**Keywords:** Cell biology, Neuroscience

## Abstract

LIM domain-binding 3 (LDB3) is a member of the Enigma family of PDZ–LIM proteins. LDB3 has been reported as a striated muscle-specific Z-band alternatively spliced protein that plays an important role in mechanosensory actin cytoskeleton remodeling. This study shows that LDB3 is broadly expressed in the central and peripheral nervous system of human and mouse. LDB3 is predominantly expressed in the adult stages compared to early development and at a significantly higher level in the spinal cord than in the brain. As in skeletal muscle and heart, LDB3 is extensively alternatively spliced in the neurons. Three novel splice isoforms were identified suggesting splicing-dependent regulation of LDB3 expression in the nervous system. Expression of LDB3 in the motor cortex, cerebellum, spinal motor neuron, peripheral nerve, and neuromuscular junction in addition to skeletal muscle indicates important roles for this PDZ–LIM family protein in motor planning and execution. Moreover, expression in the hippocampal neurons suggests roles for LDB3 in learning and memory. LDB3 interactors filamin C and myotilin are also expressed in the spinal motor neuron, nerve, and neuromuscular junction, thereby providing the basis for neurogenic manifestations in myopathies associated with mutations in these so-called muscle proteins.

## Introduction

The Enigma family of proteins is comprised of Enigma (PDLIM7), Enigma Homolog (ENH; PDLIM5), and LIM domain-binding 3 (LDB3; PDLIM6 also called ZASP, Cypher, Oracle). Enigma and ENH are expressed in multiple tissues including muscle and brain^1–3^. In contrast, LDB3 expression is described primarily in striated muscle^4,5^. Multiple LDB3 splice isoforms and their Z-disc localization have been characterized in skeletal muscle and heart^6–10^. Interestingly, LDB3 was initially detected through sequencing of a brain cDNA library^4,11^ however, the splicing spectrum and localization of LDB3 in the nervous system are not yet characterized.

As a part of the Enigma family, LDB3 contains the N-terminal PDZ domain and three LIM domains at the C-terminus^4,5^. The association of the PDZ and LIM modular protein-interacting domains allows LDB3 to act as an adaptor to recruit large molecular complexes at specific subcellular sites. Expression of LDB3-interacting proteins including α-actinin-2, filamin C, myotilin, and PKCα has already been reported in the brain^12–15^. Neurogenic findings are a part of the spectrum of degenerative diseases caused by mutations in LDB3, filamin C, and myotilin^16–19^. Moreover, LDB3-regulated proteins such as PKCα and filamin C are recognized as potential therapeutic targets in cancers and degenerative diseases of brain^10,20–23^. Taken together these observations led us to systematically examine the developmental and spatial expression of LDB3 in the mouse and validate the findings in adult human nervous system.

## Results

### Expression of LDB3 in mouse and human brain and spinal cord

**Figure 1:**
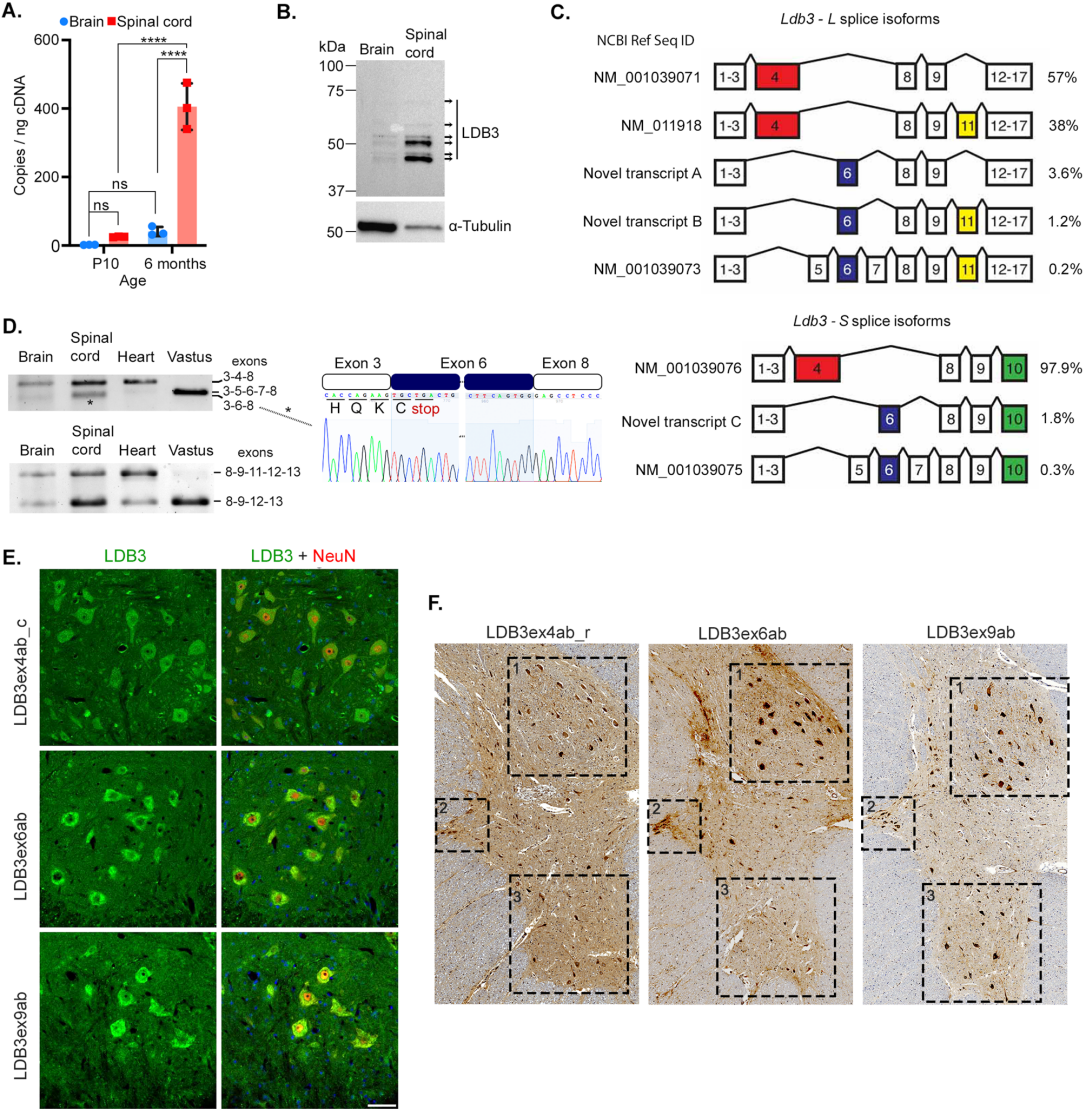
Expression of LDB3 splice isoforms in brain and spinal cord. (**A**) Expression levels of *Ldb3* mRNA in brain (blue circle) and spinal cord (red square) of 10-day (P10)- and 6 month-old mice measured with ddPCR assays using equal amounts of input cDNA per reaction. The bar graph with scatter plot represents mean ± S.D.; n = 3 mice; triplicate assay; a two-way ANOVA with the Bonferroni correction ****p < 0.0001. (**B**) A representative immunoblot of LDB3 isoforms in human motor cortex and spinal cord detected by a rabbit monoclonal antibody (Abcam; ab171936). Multiple bands are seen between 37 and 75 kDa (arrows). Two-fold higher amount of proteins was loaded from the brain lysate compared to the spinal cord; α-tubulin served as a loading control. (**C**) Spectrum of LDB3-L and LDB3-S isoforms detected by SMRT sequencing in the brain of six month old mice (n = 3). Relative percentages of total reads are shown. (**D**) Representative gel electrophoresis of *Ldb3* splice isoforms in different mouse tissues in a RT-PCR assay (n = 6 mice). Exon composition identified by Sanger sequencing is indicated. Chromatogram of a cloned amplicon from the lower band (*) in the spinal cord validates the novel splicing events joining *Ldb3* exon 3 to 6 and exon 6 to 8. Predicted reading frame including premature stop codon in exon 6 is shown. Dotted line indicates continuity of exon 6 sequence in the chromatogram. See Supplementary Table S2 for all primer sequences. (**E**) Representative immunofluorescence on lumbar spinal cord cross sections of six month old mice (n = 6) show staining for the exon domain-specific LDB3 antibodies (green) in NeuN (red)-positive neurons of the anterior horn. Scale bar: 20 µm. (**F**) Representative immunohistochemistry on serial cross sections of human L1-L2 spinal segments (n = 3 autopsies) show staining for the exon-specific antibodies in neurons of Clarke’s dorsal nucleus (1), the lateral column (2), and the anterior horn (3). Individual tiles were acquired at 20 × magnification and stitched together into a composite image. Original blots/gels for the image B and D are presented in Supplementary Fig. 2 of supplementary file 1.

Total cellular *Ldb3* mRNA levels measured by ddPCR were increased 19-fold and 16-fold, respectively from postnatal day 10 to age six months in mouse brain and spinal cord (Fig. 1A). Compared to brain, *Ldb3* mRNA levels were tenfold higher in the spinal cords of six month old mice (Fig. 1A; Bonferroni adjusted p < 0.0001). Immunoblotting of human brain and spinal cord lysates showed higher levels of LDB3 protein in spinal cord relative to brain (Fig. 1B).

### LDB3 splice isoforms in mouse and human brain and spinal cord

We consistently observed multiple bands in the immunoblots of brain and spinal cord lysates similar to that seen in muscle^4^, suggesting the presence of alternatively spliced LDB3 isoforms in the nervous system. Higher yield of total cellular RNA with RNA Integrity Number (RIN) > 9 enabled examination of the LDB3 splice isoforms with Single Molecule Real Time (SMRT) sequencing in the brain of 6 month old mice. Novel *Ldb3* splice isoforms containing exons 3–6–8 cassette were identified in long and short *Ldb3* isoform libraries (Fig. 1C; *Ldb3-L* and *Ldb3-S*, respectively; Genbank ID MH027380-MH027382). A majority of reads conformed to the annotated *Ldb3* transcripts on the Ensemble genome browser and contained exon 4 in *Ldb3-L* and *Ldb3-S* libraries. The exons 5–6–7 cassette was detected only rarely in the brain. *Ldb3* exons 4 and 6 were mutually exclusive, supporting previous observations in skeletal muscle and heart^6,7,24^. About two-thirds of *LDB3-L* reads excluded exon 11 (exon 10 in human). Next, we validated the SMRT sequencing findings with RT-PCR, Sanger sequencing, and immunofluorescence assays.

RT-PCR followed by Sanger sequencing of cloned amplicons generated using primers in canonically spliced exons 3 and 8 validated the exon 3–4–8 splicing similar to that seen in heart, the exon 3–5–6–7–8 splicing as in skeletal muscle, and novel exon 3–6–8 splicing in brain and spinal cord of 6 month old mice (Fig. 1D). LDB3 exon 3–6–8 splicing was not detected in heart or skeletal muscle. We confirmed both exon 11 (exon 10 in human) inclusion and exclusion in *Ldb3-L* isoforms in brain and spinal cord by sequencing of RT-PCR products using primers in canonically spliced exons 8 and 13 (Fig. 1D). This splicing pattern was similar to heart but contrasts with skeletal muscle in which most *Ldb3-L* isoforms excluded exon 11 (Fig. 1D).

Expression of distinct LDB3 isoforms was also examined by immunostaining with custom-made rabbit polyclonal antibodies against epitopes in alternatively spliced LDB3 exons exon 6^23^, 4 and 9 (exon 10 in mice; Supplementary Fig. 1A). These knockout validated antibodies specifically recognized corresponding LDB3 exon-encoded domains in recombinant purified LDB3 peptides, transfected cell culture systems, and in mouse vastus lateralis muscle and heart (Supplementary Fig. 1B–E^23^). Immunofluorescent staining with these validated antibodies showed LDB3 isoforms containing exon 4-,6- and 10-encoded domains in the cell bodies and proximal processes of anterior horn neurons in the spinal cord of 6 month old mice (Fig. 1E). Further, immunohistochemical staining with these antibodies detected distinct LDB3 isoforms in the nucleus dorsalis (Clarke’s nucleus) neurons in the posterior horn, preganglionic sympathetic neurons in the lateral horn, and the anterior horn neurons in the human spinal cord L1–L2 sections (Fig. 1F).

### Spatial expression of LDB3 in the mouse brain

**Figure 2:**
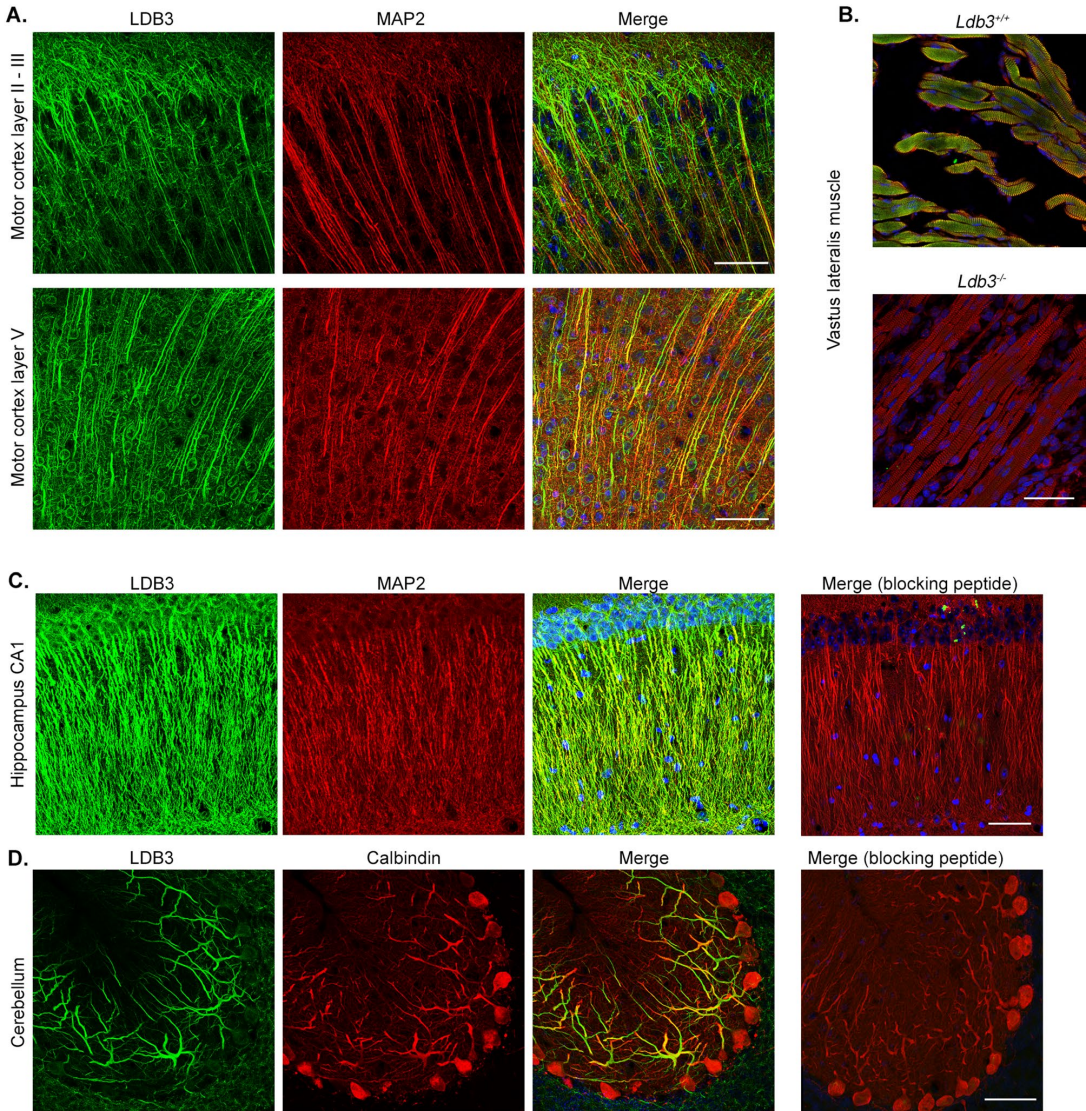
Immunolocalization of LDB3 in different regions of mouse brain. (**A**) Representative immunofluorescence using GTX115593 antibody on motor cortex coronal section shows LDB3 signal (green) in the apical dendrite bundles probably from layers III and V stained by MAP2 (red) and in thinner dendrites not labeled by MAP2 which are likely from superficial neurons in six-month old mice (n = 6). Bottom panel shows LDB3 signal (green) in MAP2 (red) stained cell body and dendrites of pyramidal neurons in motor cortex layer V in the same coronal section. (**B**) Representative immunofluroscence shows the same LDB3 antibody signal (green) co-localized with α-actinin-2 signal (red) at the Z-discs in the vastus lateralis muscle fibers of 18 day old *Ldb3*^+*/*+^ embryo but not *Ldb3*^*−/−*^ littermates (n = 3). Note *Ldb3*^*−/−*^ mice do not survive beyond birth. (**C**, **D**) Representative immunofluorescence using the same antibody shows LDB3 (green) in MAP2 (red) stained dendrites from the hippocampal CA1 neurons (**C**) and in calbindin (red) stained dendrites from the cerebellar Purkinje cells (**D**) in 6 month old mice (n = 6). LDB3 signal is absent in parallel sections incubated with LDB3 antibody plus a blocking peptide. Scale bar in all images 50 µm.

We examined LDB3 expression in functionally different brain regions including the motor cortex, hippocampus, and cerebellum of 6 month old mice. For this purpose we used previously published LDB3 antibodies in immunofluorescence assays^8,23,25^. These antibodies recognized both LDB3-L and LDB3-S isoforms in immunoblotting assays (Supplementary Fig. 1B,C). LDB3 immunostaining was detected in the soma and dendrites of neurons in the motor cortex layers II, III, and V (Fig. 2A). The target specificity of the antibody was confirmed by staining the vastus lateralis muscle of *Ldb3*^+/+^ and *Ldb3*^−/−^ littermates on the same slide as the brain tissues (Fig. 2B). We also found LDB3 in the CA1 pyramidal neurons of the hippocampus (Fig. 2C). In the cerebellar cortex, LDB3 staining was mostly localized to the dendritic arbors of Purkinje cells (Fig. 2D). LDB3 staining was absent after blocking the antibody by incubating with purified untagged LDB3 peptide, thus validating the antibody specificity for LDB3 protein in neurons.

### LDB3 expression in human motor cortex and spinal cord

**Figure 3:**
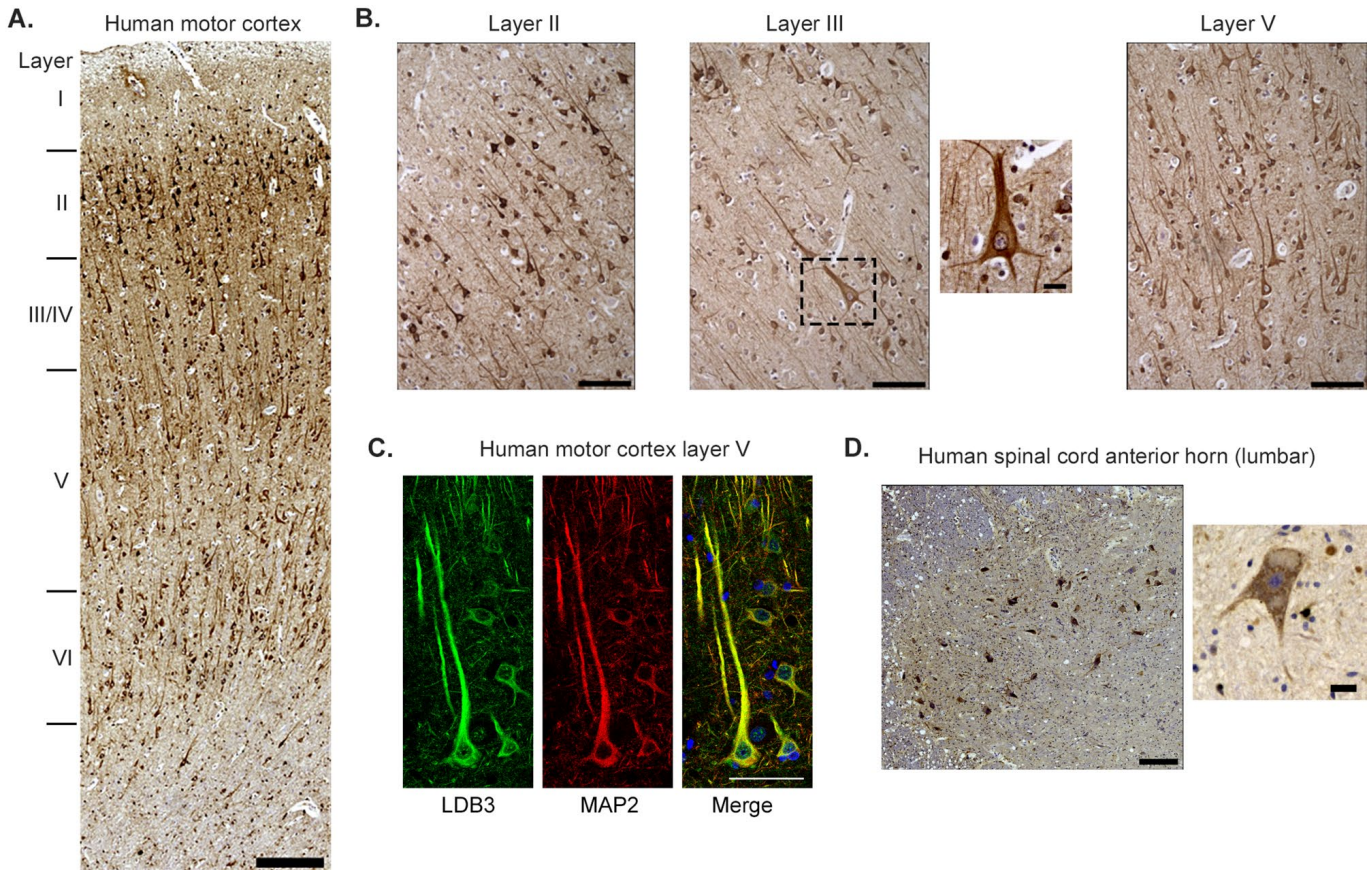
Immunolocalization of LDB3 in human motor cortex and the L1-L2 spinal cord anterior horn. (**A**) Immunohistochemistry staining with GTX115593 antibody shows LDB3 immunoreactivity in neurons of the motor cortex layers. (**B**) Images show neurons of layers II, III, and V at a higher resolution. The LDB3 signal is seen in the soma and processes of the neurons. (**C**) Immunofluorescent staining with the same antibody shows LDB3 (green) in the soma and dendrites of MAP2 co-stained (red) pyramidal neurons of layer V in the motor cortex. (**D**) Immunohistochemistry staining with the same antibody shows LDB3 immunoreactivity in the soma and processes of the anterior horn neurons in lumbar segments of the spinal cord. Scale bar 250 µm (**A** and **D**), 100 µm (**B**), 50 µm (**B** inset and **C**), and 20 µm (**D** inset).

Next, we examined the expression of LDB3 in coronal sections of human motor cortex and spinal cord L1–L2 axial sections from autopsy by immunostaining with a knockout validated antibody. Immunohistochemical staining detected LDB3 in neurons from layers II to VI of the human motor cortex (Fig. 3A). As in mice, LDB3 immunoreactivity was seen in the soma and processes of neurons in layers II, III, and V (Fig. 3B). Immunofluorescence studies confirmed the presence of LDB3 in the MAP2-stained cell body and dendrites of the pyramidal neurons including the gigantic Betz cells in layer V of the human motor cortex (Fig. 3C). In L1–L2 human spinal cord, LDB3 immunoreactivity was seen in the soma and processes of the anterior horn neurons (Fig. 3D).

### Expression of LDB3 and its interactors filamin C and myotilin in the peripheral nervous system

**Figure 4:**
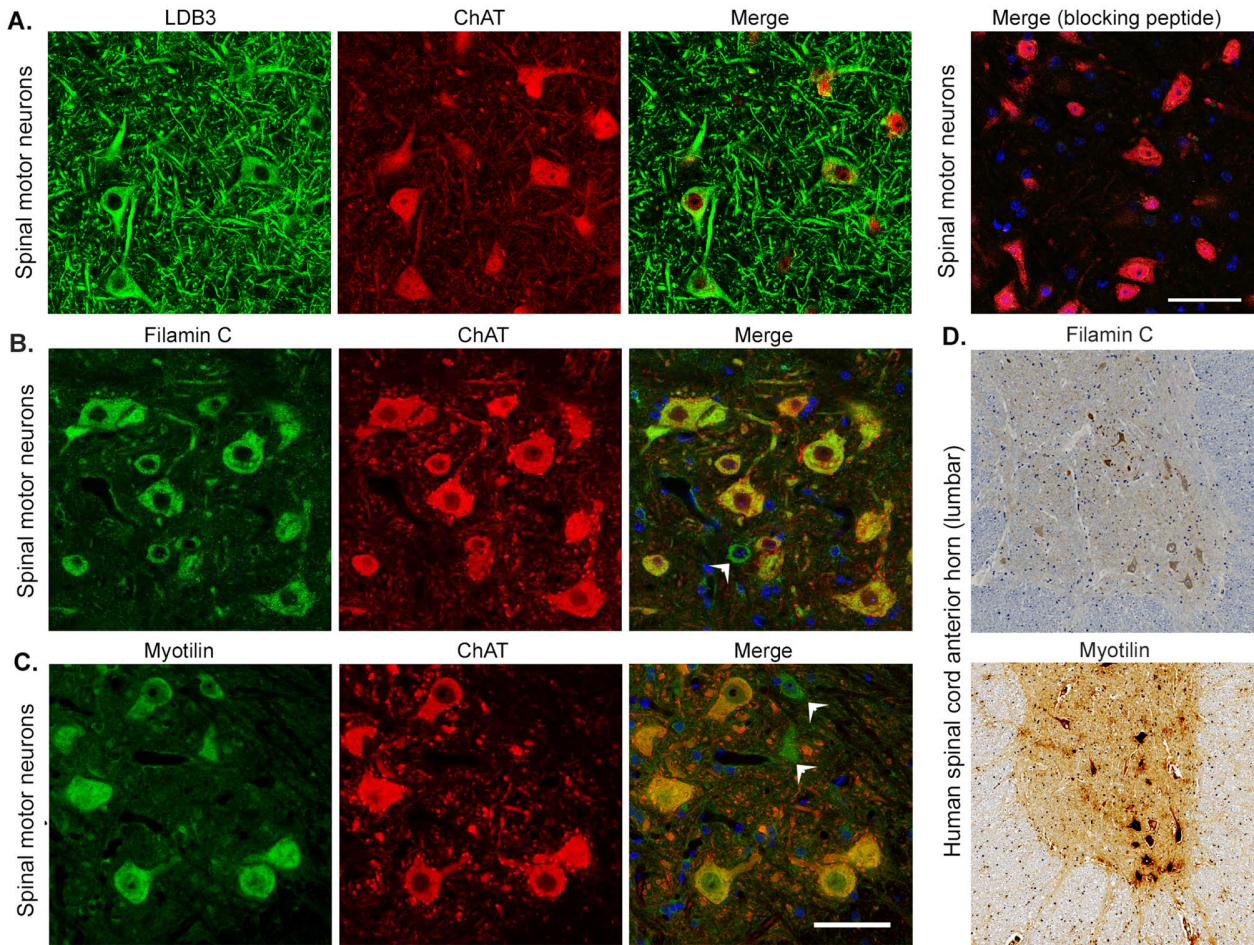
Immunolocalization of LDB3, filamin C and myotilin in spinal motor neurons. (**A**) Representative immunofluorescence on spinal cord lumbar cross section stained by GTX115593 antibody show LDB3 signal (green) in the cell body and processes of ChAT-positive (red) spinal motor neurons of six month old mice (n = 6). Addition of a blocking peptide results in loss of the LDB3 signal. (**B**, **C**) Immunostaining of parallel sections with antibodies against filamin C (**B**) and myotilin (**C**) detect both proteins in cholinergic neurons in the anterior horn of the spinal cord in mice (n = 6). Arrow heads indicate myotilin- and filamin C -antibody stained cells which are not ChAT positive. (**D**) Representative immunohostochemistry on human spinal cord lumbar segments (n = 3; 20 × magnification) show that neurons in the anterior horn react to the filamin C and myotilin antibody. Scale bar: 50 µm.

**Figure 5:**
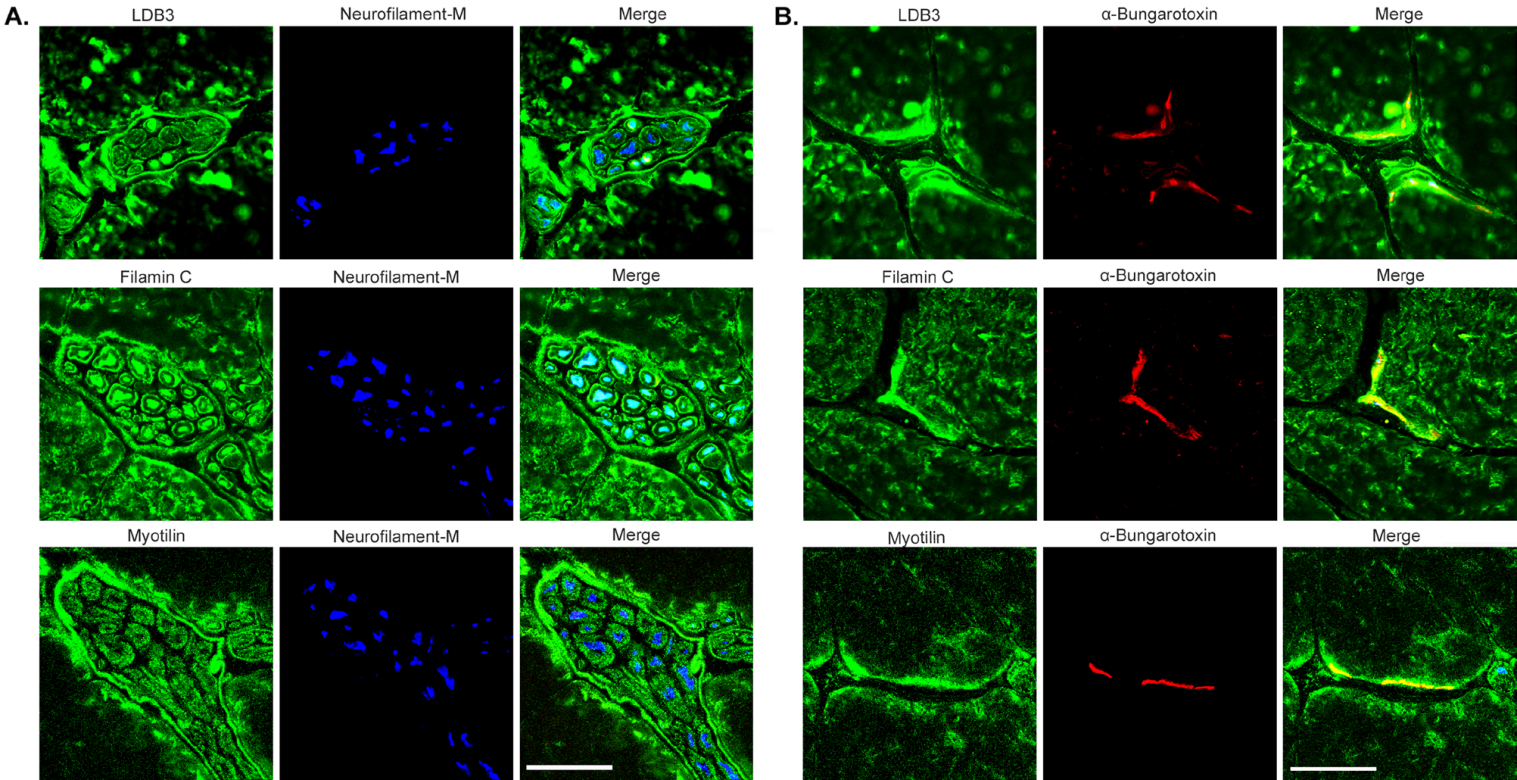
Immunolocalization of LDB3, filamin C, and myotilin in peripheral nerve and NMJ. (**A**, **B**) Representative immunofluorescence on tibialis anterior muscle cross-sections from six-month old mice (n = 6) show LDB3, filamin C, and myotilin staining (green) localized to intramuscular nerve fibers (**A**) and NMJs (**B**) marked by expression of neurofilament-M (blue) and α-Bungarotoxin (red), respectively. Scale bar (25 µm).

In the anterior horn of the spinal cord, cholinergic motor neurons are unique neuronal cells connecting the brain to the periphery through long axons terminating in neuromuscular junctions (NMJs) in skeletal muscles. Lower motor neuron syndrome and axonal neuropathy have been reported in patients with mutations in LDB3, filamin C, and myotilin^16,17,19^. We asked whether LDB3 and its interacting proteins filamin C and myotilin are expressed in the cholinergic motor neurons of the spinal cord, peripheral nerves, and NMJs. We detected LDB3 in the soma and processes of ChAT positive motor neurons in the anterior horn of the spinal cord from 6 month old mice (Fig. 4A). The antibody signal was absent in the sections incubated with blocking peptide as in the brain. Staining of parallel spinal cord sections showed filamin C and myotilin immunoreactivity in the ChAT positive motor neurons (Fig. 4B,C). As with LDB3 (Fig. 3D), immunohistochemical assays detected filamin C and myotilin antibody stained anterior horn neurons in human L1–L2 spinal cord axial sections (Fig. 4D). LDB3, filamin C, and myotilin were detected in intramuscular nerves and NMJs in the tibialis anterior muscle of six month old mice (Fig. 5A,B).

## Discussion

LDB3 has thus far been regarded as a muscle-specific protein that plays an important role in maintaining the actin cytoskeleton integrity in multiple species^26–28^. The present study demonstrates the developmental and spatial expression of LDB3 in the mouse and human nervous system. The precedence for expression of a presumed muscle-specific LIM protein in the nervous system was established by Muscle LIM Protein which was subsequently shown to promote neuroregeneration^29,30^. Similar to muscle, multiple splice isoforms of LDB3 exist in the nervous system including three as yet unidentified isoforms. LDB3 is predominantly expressed in the cell body and dendrites of neurons during adult stages of mouse brain and spinal cord. Relative to brain, LDB3 expression levels are significantly higher in spinal cord. In particular LDB3 is expressed in the upper motor neurons, cholinergic lower motor neurons, peripheral nerve, and NMJ that effectively regulate muscle contraction. Moreover, we found that LDB3 is expressed in the cerebellar Purkinje cells regulating coordination and learning of movements, and the hippocampal CA1 pyramidal neurons fundamental to learning and formation of memories.

In terms of anatomical localization and agility, the vulnerability of spinal cord to undergo a greater mechanical stress as compared to brain may require more frequent repair of cytoskeletal proteins for stabilization of cellular function^31^. Increased reactive oxygen species production in mitochondria of spinal cord as compared to brain leads to greater propensity of oxidative stress in motor neurons of the spinal cord^32^. There is in-vitro evidence that most neuronal and glial cell types adapt their morphology and cytoskeletal configuration to mechanical stimuli in their environment^33^. LDB3 is a cytoskeleton associated protein and binds to mechanosensitive region of cytoskeletal proteins linked to actin and some known heat shock proteins (HSPs) thus functions as a multivalent interaction hub contributing in cellular mechanosensing^23^. In this background, the underlying reason for a significantly high LDB3 expression in spinal cord compared to the brain in adult mice could be a cellular counterintuitive strategy to deal with greater mechanical stress in spinal cord. Like our findings, a significantly higher expression of HSPs also interact and co-operate with LDB3^23^, was reported in spinal cord as compared to brain in wild type mice and in multiple sclerosis patient tissue^31,34^. HSPs interact with the cytoskeleton to avoid aggregation, support assembly, and facilitate stabilization of cytoskeletal proteins. The underlying reason for the high HSPs was attributed to differences in astrocyte cytoskeleton composition and to a subset of astrocytes and microglia with higher HSP expression in spinal cord and cortical primary mixed glial cultures^31,34^. Broader expression of LDB3 in distinct regions of the brain and spinal cord supports a widespread role of this PDZ-LIM protein such as in planning and executing motor function and higher level functions like learning and memory.

We applied long-read sequencing technology to map alternative splice sites within single *Ldb3-L* and *Ldb3-S* transcripts in the nervous system. A large majority of isoforms in the mouse nervous system can be explained by alternative splicing at sites previously reported in studies of heart and skeletal muscle^6,7,24^. In mouse, *Ldb3* isoforms with exon 4 were known to be expressed only in the heart^7^. Here, we show that the *Ldb3-L and Ldb3-S* isoforms with exon 4 are also predominent in brain and spinal cord. In contrast, skeletal muscle-specific *Ldb3* isoforms containing the exon 5–6–7 cassette were only rarely detected in the brain. The novel *Ldb3* splice isoforms with the exon 3–6–8 cassette in the brain and spinal cord were absent in skeletal muscle and heart. Nonsense-mediated mRNA decay likely affected the transcript levels as joining of exon 3 to 6 shifts the reading frame resulting in a premature termination codon within exon 6. We generated and validated new domain-specific antibodies which identified specific LDB3 isoforms in mouse and human skeletal muscle, heart, and neurons. These antibodies will be useful in studies of LDB3 isoform-specific roles in different tissues. RT-PCR assays and immunofluorescence with isoform-specific antibodies detected expression of LDB3 exon 4, 6, and 10 (exon 9 in human) in neurons, which contain hotspots for rare mutations causing skeletal and cardiac myopathy^17,24^. In addition to LDB3, other rare myopathy gene products such as filamin C and myotilin are expressed in the nervous system [this study; (Mologni^13^; Xie^14^)]. Clinical studies are needed to better define the natural history and full spectrum of disease presentation in patients with LDB3, filamin C, and myotilin mutations.

Multiple LDB3 splice isoforms enable heterogeneous protein interactions, modify mutation effects and alter disease severity^8,23,35^. The PDZ domain is present in all LDB3 isoforms, whereas LIM domains are only present in *Ldb3-L* splice isoforms^6,7,24^. We identified both *Ldb3-L* and *Ldb3-S* isoforms in the nervous system. The *Ldb3-L* isoforms negatively regulate PKCα expression^10^. LDB3 avidly binds to actin and facilitates mechanosensing-directed actin cytoskeleton remodeling^8,9,23^. As an A-kinase anchoring protein, LDB3 plays a role in β-adrenergic stimulation of cardiac Ca_v_1.2 channels^36^. Interestingly, the spatial distribution of LDB3 mirrored that of MAP2, a dominant A-kinase anchoring protein in neurons^37^. These LDB3 functions are not unlike other PDZ scaffold proteins, for example postsynaptic density protein 95, which facilitate functional coupling of receptors and ion channels to downstream signaling pathways in synapses and neurons^38^. More recent studies indicate the role of activity dependent actin cytoskeleton remodeling in synapse-specific delivery and plasticity^39^. Whereas LDB3 is detectable in myocytes from early embryonic stages to adulthood^5^, we found that *Ldb3* expression in the mouse nervous system occurs preferentially during adult stages. In agreement with previous observations^28^, LDB3-deficient mice did not survive after birth which limited their use to examine the role of LDB3 in the adult nervous system. While outside the scope of this study, conditional gene knockout model systems are necessary to solve the enigma of LDB3 functions in the central and peripheral nervous system during specific stages of development.

## Conclusion

In summary, our findings indicate that LDB3 is expressed broadly in the adult central and peripheral nervous system of mouse and human. The splicing pattern of LDB3 in the brain and spinal cord is more similar to that of heart than skeletal muscle. The expression of LDB3 and interacting proteins in the nervous system indicates expansion of clinical phenotype and genotype relationship in degenerative diseases. Importantly, LDB3 has roles in regulating activity-dependent actin cytoskeleton remodeling and its presence in the neurons should stimulate investigations for novel mechanisms of neuronal trafficking and synaptic plasticity.

## Materials and methods

### Mice

Wild type mice (C57BL/6N) were from Charles River Laboratories (Wilmington, MA). *LDB3*^+*/−*^ mice (C57BL/6N-A^tm1Brd^/a*Ldb3*^*tm2a(EUCOMM)Hmgu*^/BcmMmucd) were from the Mutant Mouse Resource & Research Center^40^. All mice were housed in the animal care unit of the National Institute of Neurological Disorders and Stroke (NINDS) according to the National Institutes of Health animal care guidelines. All animal and human studies and experimental protocols included in the present manuscript were approved by the National Institutes of Health Institute/Center Animal Care and Use Committee (Animal study protocol—1355). The present study is reported in accordance with ARRIVE guidelines.

### Postmortem human tissues

The analyzed formalin-fixed paraffin embedded (FFPE) human samples were derived from different individual postmortem and obtained from the University of Miami Brain Endowment Bank (Institutional Review Board protocol number 19920358 (CR0001775). The present study is reported in accordance with ARRIVE guidelines.

### Antibodies

Primary antibodies used in this study are in Supplementary Table 1 (Supplementary file 1). Alexa Fluor- and HRP-conjugated secondary antibodies were purchased from Invitrogen and Jackson Immunoresearch Laboratories, respectively. Alexa Fluor-555 conjugated α-Bungarotoxin (α-Btx; B35451; Life Technologies) was used as a marker for acetylcholine receptors at the NMJ in skleletal muscle.

### DNA constructs

GFP-tagged human LDB3-S constructs were described previously^23^. Primers are listed in Supplementary Table 2 (Supplementary file 1).

### Purified LDB3 proteins

Protein expression and purification of His-tagged and untagged LDB3 peptides have been described elsewhere^9^.

### RNA isolation from tissue

Total cellular RNA was isolated using TRIzol Reagent (Life Technologies) followed by on-column DNase I digestion and purification with RNeasy Mini Kit (Qiagen). RNA with greater than 8 RIN (Agilent 2100 Bioanalyzer) were used for all assays. RNA was stored at − 80 °C.

### Reverse transcription: polymerase chain reaction (RT-PCR)

RT was done in 20 µl volume using 1 µg total cellular RNA and SuperScript III reverse transcriptase (Invitrogen) with oligod(T)s and random hexamers. The cDNA samples were stored at − 20 °C. The cDNA was quantified using the ssDNA OliGreen Quant-iT kit (Thermo Scientific). PCR primers flanking alternatively spliced *Ldb3* exons are described in Supplemental Table 2 (Supplementary file 1). PCR products were resolved on agarose gels, stained with GreenGlo (Denville, Holliston, MA) and analyzed on a ChemiDoc XRS + Molecular Imager (Bio-Rad). These agarose gel bands were excised and isolated cDNA was either directly analyzed by Sanger sequencing or cloned into pCR2.1 plasmid by TOPO-TA cloning (Life Technologies) and sequenced from individual colonies.

### Droplet digital PCR (ddPCR)

ddPCR assays were run on Bio-Rad QX200 system (Bio-Rad, Hercules, CA) using TaqMan probes spanning constitutively spliced exons 2 and 3 (Mm01208763_m1; Life Technologies), thus allowing measurement of total *Ldb3* transcript levels. Equal amounts of input cDNA (5 ng) were used per reaction and values are presented as copies per ng of input cDNA. Multiple biological replicates were used in each assay (n ≥ 3). At least three independent assays were performed for each sample. Data were analyzed using the QuantaSoft software (Bio-Rad). The relative LDB3 expression in mouse brain regions (mouse cortex, hippocampus, basal ganglia, and cerebellum) during postnatal development stage P0, P4 and P8 was very low/negligible hence P10 was used as a starting reference point to compare with the adult stage (6 month) for ddPCR based quatification.

### Single molecule real time sequencing and analysis

cDNA was synthesized from RNA samples with RIN values of ≥ 9.8 using the SMARTer PCR cDNA synthesis kit (634925; Clontech, Mountain View, CA) and amplified using PCR primers specific for *Ldb3-L* and *Ldb3-S* isoforms (Supplemental Table 2; Supplementary file 1). PCR products were purified using AMPure® PB magnetic beads (100-265-900; Pacific Biosciences, Menlo Park, CA) and quantified/sized on an Agilent 2100 Bioanalyzer. Approximately 500 ng of each sample was used to generate SMRTbell libraries and the polymerase-bound complexes were sequenced using a PacBio RS II and v3 SMRT Cells (100-171-800; Pacific Biosciences). Sequencing results were aligned to genomic sequence (Ensembl GRCm38.p3) by the ultra-fast universal RNA-seq aligner STAR (https://github.com/alexdobin/STAR/releases). The RNA-seq alignment program STAR v2.3.1 and the in-house MatchAnnot program (https://github.com/TomSkelly/MatchAnnot) were used to map full-length non-chimeric reads to the *Ldb3* gene locus (mouse reference version GRCm38 and GENCODE annotation M8). Reads with < 2 base pairs (bp) mismatch were categorized as fully matched and for the remaining mapped reads, an exclusion threshold was set at a count support of < 1%. We were able to map 24,719 *Ldb3-L* and 10,027 *Ldb3-S* full length, non-concatemer reads (average read length = 2875 bp and 963 bp, respectively). Of these reads, 89.7% and 94.3% were considered above set threshold, respectively. Ldb3 mapped reads were classified as known transcripts or novel splice variants based on GENCODE annotation by MatchAnnot program. Manual inspection by Integrative Genomics Viewer (http://software.broadinstitute.org/software/igv/) defined the exon makeup of the *Ldb3* reads.

### Tissue samples and preparation

Hindlimb muscles and heart from mice were prepared by snap-freezing in liquid nitrogen-chilled isopentane. Brain and spinal cord tissues were collected from mice perfused with 4% paraformaldehyde, post-fixed in 4% paraformaldehyde overnight, and later equilibrated in 30% sucrose overnight. FFPE human motor cortex and lumbar spinal cord (L1–L2) tissues were from individuals over 18 years of age with no indicated neurological disorders. Human spinal cord whole tissue lysates (NB820-59258) were purchased from Novus Biologicals (Centennial, CO).

### Immunofluorescence and immunohistochemistry

Immunofluorescence assays were done on floating 50 µm brain and spinal cord sections, and 9 µm skeletal muscle sections placed onto charged slides. Immunohistochemistry assays were done on 5 µm brain and spinal cord sections placed onto charged slides. Tissue sections were stained with primary antibody at 4 °C for 16 h, washed 5 times in phosphate-buffered saline for 2 min each and then stained with secondary antibody for 1 h at room temperature. A Leica BOND-MAX Automated Stainer (Leica Biosystems, Buffalo Grove, IL) with epitope retrieval of Citrate pH 6.0 for 20 min was used for immunohistochemistry. We examined at least 3 different dilutions of each antibody and pre-immune sera or blocking peptide when available. Antibody was blocked by incubation with LDB3 peptide at a ratio of 10:1 for 1 h at 4 °C before staining for signal validation. During each of the assays, negative controls were always processed following the same protocol with the omission of the primary antibody to evaluate any non-specific labeling. Immunofluorescence images were acquired using a Leica confocal microscope (TCS SP5 576 II) equipped with set of a 40 ×/NA 1.3 oil immersion Plan-Apochromat objective lens at 1024 × 1024 resolution. Tile scans were acquired (20 ×) using an Axio Scan.Z1 slide scanner (Zeiss) and stitching was done using the ZEN Blue software (Zeiss). All the image acquisition and display settings were the same between groups and comparisons were made for tissue sections stained on the same slide. The images were adjusted for appropriate size, correction of brightness, contrast, intensity and presentation in figures at resolution 300, using Adobe Photoshop CC 20.0.10 Release (Adobe systems, San Jose, CA). All images are shown with the representative scale.

### Transfections

COS-7 cells were transfected using Lipofectamine 2000 reagent (Life Technologies) to express C-terminal GFP-tagged full-length human LDB3-S isoforms containing either exon 4- or exon 6-encoded sequences (NP_009009.1 and NP_001073585.1, respectively).

### Immunoblotting

Protein samples were separated by polyacrylamide gel electrophoresis and transferred to PVDF membranes (Life Technologies). After blocking in 5% low fat milk for 1 h at room temperature, the membranes were incubated with primary antibody overnight at 4 °C and then with HRP-conjugated secondary antibodies (Jackson ImmunoResearch Laboratories) for 1 h at room temperature. The ChemiDoc XRS + Molecular Imager (Bio-Rad, Hercules, CA) was used for detection. The blots were cropped or adjusted for appropriate size using Adobe Photoshop CC 20.0.10 Release (Adobe systems, San Jose, CA) for presentation in figures. The original blots are shown in Supplementary File 1 (Supplementary Fig. 2).

### Statistical analysis

Statistical analyses were done using GraphPad Prism 8. A two-way ANOVA using the Bonferroni’s multiple comparison test was used to determine differences in *Ldb3* expression between tissue and between age. Statistical significance was defined as *p* < 0.05.

## Supplementary Information

Supplementary Information.

## Data Availability

The data generated during and/or analyzed during the current study are available from the corresponding author on reasonable request. Raw data of SMRT sequencing analysis is publically available in The National Center for Biotechnology Information (NCBI) SRA database repository https://www.ncbi.nlm.nih.gov/sra/?term=PRJNA436964. The sequences of novel *Ldb3* isoforms are available in GenBank, the NIH genetic sequence database (ID: MH027380-MH027382; https://www.ncbi.nlm.nih.gov/nuccore/MH027380—https://www.ncbi.nlm.nih.gov/nuccore/MH027382). Details of antibodies and primers used in the current study are in Supplemetary Tables 1 and 2, respectively in Supplemntary File 1. Full length gels and blots are shown in Supplementary Fig. 2 in Supplementary File 1.
