# Supplementary material for: Expression of LIM domain-binding 3 (LDB3), a striated muscle Z-band alternatively spliced PDZ-motif protein in the nervous system
Source: Sci Rep. 2023 Jan 6;13:270. doi: 10.1038/s41598-023-27531-5 (PMC9822979; doi:10.1038/s41598-023-27531-5)
Supplement: Supplementary file 1 — Supplementary Information. [file 41598_2023_27531_MOESM1_ESM.pdf]

Supplementary Figure 1

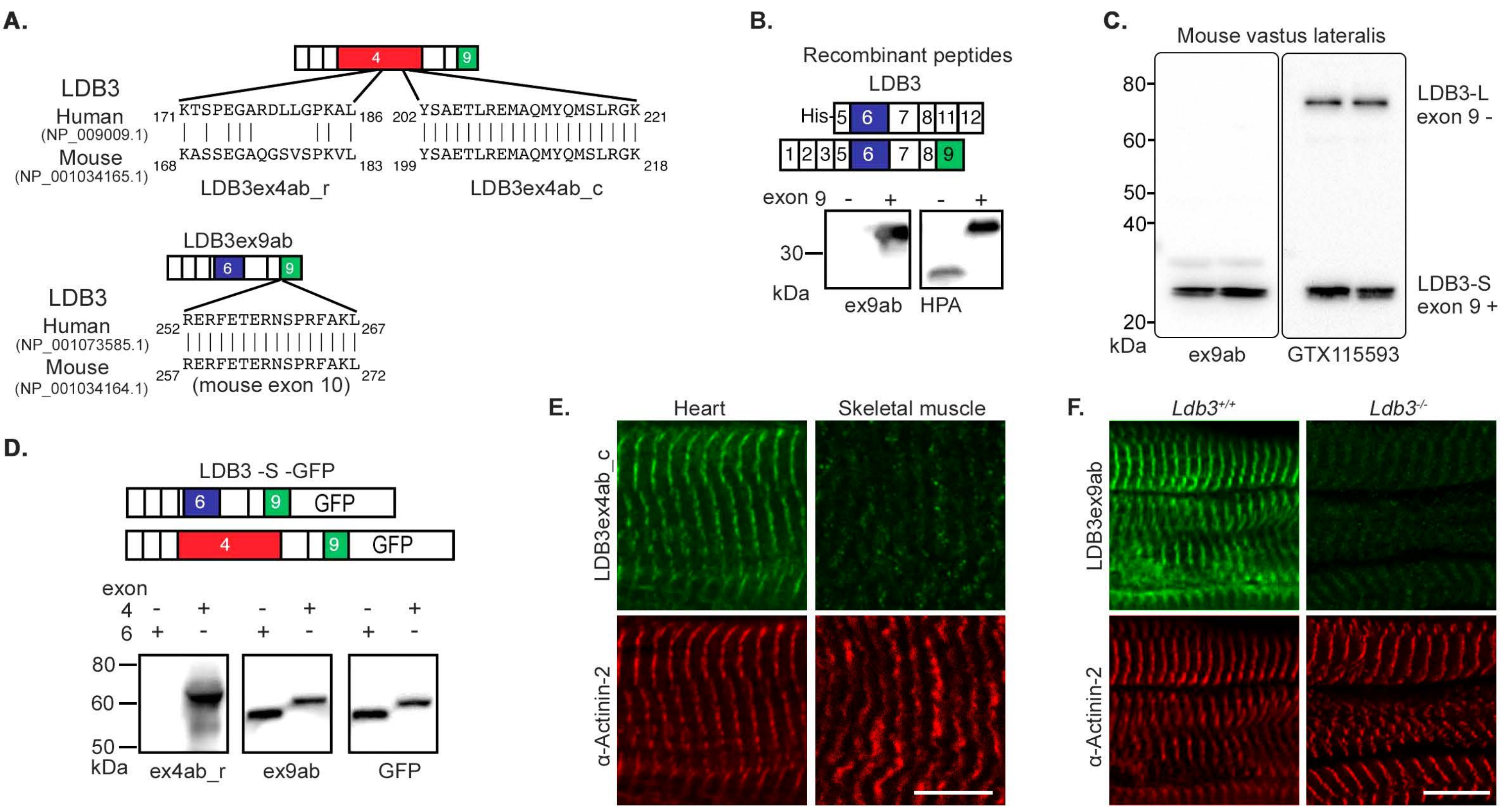

Supplementary Figure 1. Validation of custom-generated LDB3ex4ab and LDB3ex9ab antibodies. A. Epitopes for rabbit polyclonal antibodies (LDB3ex4ab_r and LDB3ex9ab) and chicken polyclonal antibody (LDB3ex4ab_c) are shown in human and mouse LDB3 proteins. Note: the epitope for LDB3ex9ab is in mouse exon 10. **B.** Immunoblots of recombinant His6-LDB3-L (exon 9 absent) peptide and untagged LDB3-S (exon 9 present) show that LDB3ex9ab specifically detects LDB3-S. In contrast HPA048955 antibody (Atlas Antibodies, Bromma, Sweden) detects both LDB3-L and LDB3-S isoforms. **C.** Immunoblot shows that LDB3ex9ab antibody selectively detects LDB3-S, whereas GTX115593 antibody (GeneTex, Irvine, CA) detects both LDB3-L and LDB3-S in the vastus lateralis muscle of six month old mice (n = 6). **D.** Immunoblots show that LDB3ex4ab_r antibody detects exon 4- but not exon 6- specific human LDB3-S isoform in transfected COS-7 cells. Note that LDB3ex9ab antibody detects both exon 4 and 6 containing LDB3-S isoforms. GFP antibody was used as a control. **E.** Representative immunofluorescence on frozen vastus lateralis and cardiac muscle longitudinal sections of six month old mice (n = 3) show staining for LDB3ex4ab_c antibody (green) co-localized with the Z-disc marker actinin (red) in the cardiac but not in skeletal muscle fibers. It is known that LDB3 exon 4 is primarily expressed in the heart 7. **F.** Vastus lateralis muscle longitudinal sections show immunofluorescent staining for LDB3ex9ab antibody (green) co-loalized with actinin2 (red) in 18 day old Ldb3+/+ but not Ldb3-/- embryo (n = 3). Note: Ldb3-/- mice died immediately after birth or were stillborn, as previously published 28. Supplementary Figure 1 and original blots for the image B, C and D are presented in Supplementary Figure 2 of supplementary file 1.

Supplementary Figure 2

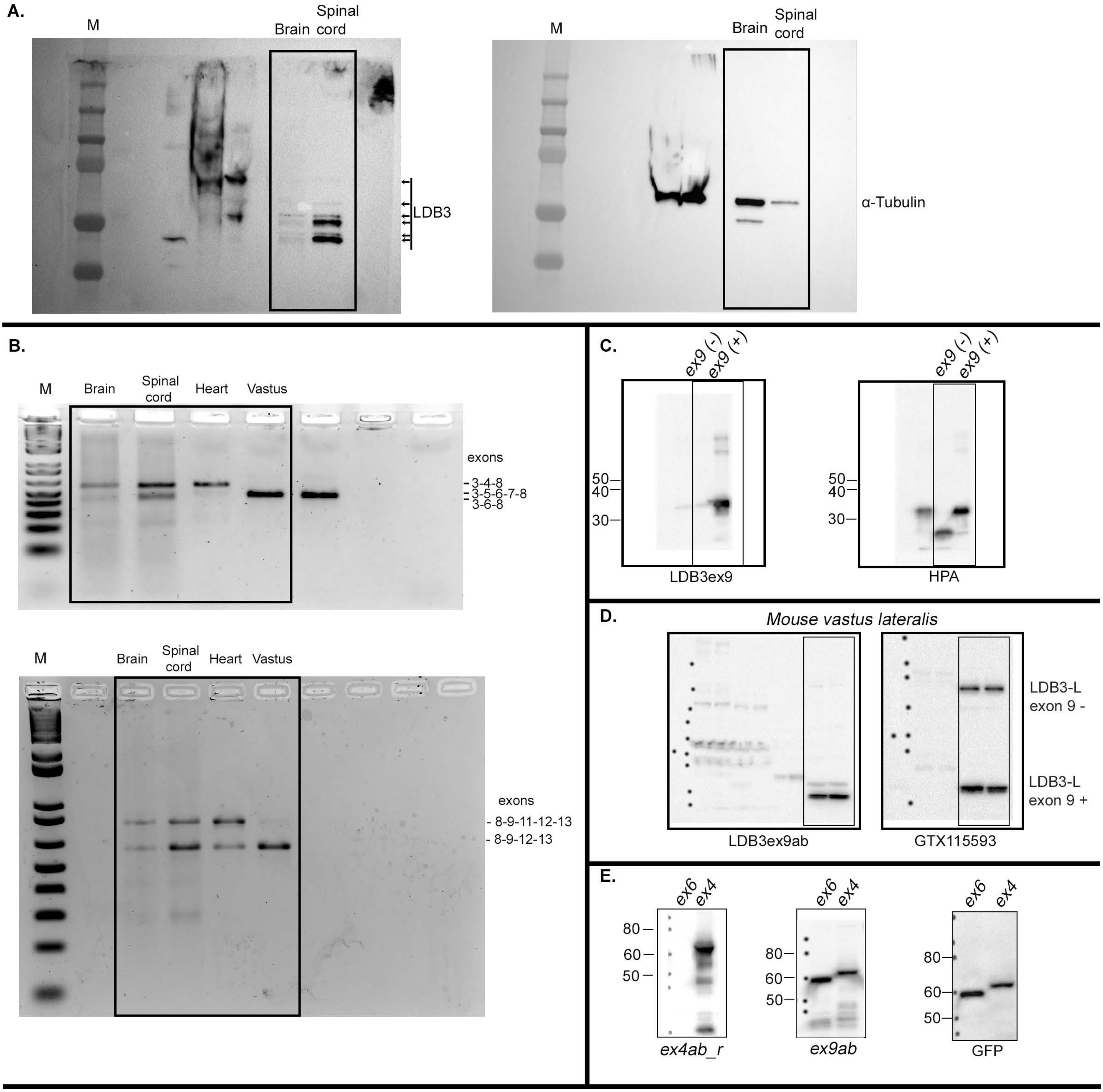

Supplementary Figure 2. Full-length Western blot and PCR gel images

A, Full-length blot images for figure 1B; **B,** Full-length gel images for figure 1D; **C,** Full-length blot images for Supplementary figure 1B; **D,** Full-length blot images for Supplementary figure 1C; **E,** Full-length blot images for Supplementary figure 1D. M = marker lane.

Supplementary Table 1. List of primary antibodies.

Protein	Species	Dilution	Application	Company	Catalog number	Reference (Pubmed ID)
LDB3	Rabbit	1: 200, 1: 1000, 1: 10,000	IHC, IF, WB	Custom-made; epitopes in exon 4, 6, and 9 (10 in human)		33742095; present study
	Chicken	1: 200	IF	Custom-made; epitope in exon 4		Present study
	Rabbit	1: 200, 1: 200, 1: 1000	IF, IHC, WB	Genetex	GTX115593	12499364
	Rabbit	1:1000	WB, IF	Atlas	HPA048955	33742095
	Rabbit	1:1000	WB	Abcam	ab171936	Present study
α-Actinin	Mouse	1: 50	IF	Sigma	EA53-A7811	20858595
α-Tubulin	Mouse	1: 1000	WB	Sigma	T6199	33627639
Calbindin	Mouse	1: 200	IF	Abcam	ab82812	33054173
ChAT	Goat	1: 20	IF	Millipore	AB144P	25485757
Filamin C	Rabbit	1: 50	IHC, IF	Novus Biologicals	NBP1-89300	33742095
GFP	Mouse	1:1000	WB	Life Technologies	MA-15256	28831037
Myotilin	Rabbit	1: 200, 1:300	IHC, IF	Abcam	ab68915	33742095
MAP2	Chicken	1: 500	IF	Abcam	ab5392	27114033
NeuN	Mouse	1:1000	IF	Millipore	MAB377	32404936
NF-M	Mouse	1:1000	IF	BioLegend	837801	31444548

Supplementary Table 2. List of *Ldb3* cDNA Primers.

Assay	Primers	Location[1]
ddPCR[2]	CAACATGCCCCTCACTATCTC<br>CCAGGTCTCCTTGGCTGAG	Exon 2<br>Exon 3
SMRT[3]	CAACATGCCCCTCACTATCTC<br>AACCTTTCCCTTGACCTTCGC	Exon 2<br>Exon 10
	AAGTCCAAGCGGCCTATTCC<br>CCATGCATTCTTCCTGAGGTTAGC	Exon 2<br>Exon 17
RT-PCR<br>Sanger sequencing	AAGTCCAAGCGGCCTATTC<br>CAGGATGCGGAAGGAGCGAGAC	Exon 3<br>Exon 8
	GGAAGATGAGGCTGATGAGTGG<br>TGCTGACAGTGGTAGTGCTCTTTC	Exon 8<br>Exon 13

[1]Refer Fig. 1C for exon numbering; [2]Droplet digital PCR; [3]Single Molecule Real Time sequencing
